# Duodenojejunostomy for endoscopic management of biliary enteric anastomotic stricture inaccessible via balloon-assisted endoscopy: a case report

**DOI:** 10.1186/s40792-023-01654-3

**Published:** 2023-05-18

**Authors:** Shinya Sakamoto, Kenta Sui, Motoyasu Tabuchi, Takehiro Okabayashi

**Affiliations:** Department of Gastroenterological Surgery, Kochi Health Sciences Center, 2125-1 Ike, Kochi-City, Kochi 781-8555 Japan

**Keywords:** Biliary stricture, Duodenojejunostomy, Biliary enteric anastomoses

## Abstract

**Background:**

Stricture formation is a long-term complication of biliary enteric anastomosis (BEA). BEA stricture often causes recurrent cholangitis and lithiasis, can significantly affect quality of life, and promote the development of life-threatening complications. In this report, duodenojejunostomy and subsequent endoscopic management as an alternative surgical technique for strictures of the BEA is described.

**Case presentation:**

Case 1: An 84-year-old man who underwent left hepatic trisectionectomy for hilar cholangiocarcinoma 6 years prior presented with fever and jaundice. Computed tomography (CT) revealed intrahepatic lithiasis. The patient was diagnosed with postoperative cholangitis secondary to intrahepatic lithiasis. Balloon-assisted endoscopy could not reach the anastomotic site, and stent insertion failed. A biliary access route was hence created via duodenojejunostomy. After the jejunal limb and duodenal bulb were identified, duodenojejunostomy was performed using a side-to-side continuous layer-to-layer suture. The patient was discharged without serious complications. Endoscopic management through duodenojejunostomy was successfully performed, and intrahepatic stones were completely removed.

Case 2: A 75-year-old man who underwent bile duct resection for hilar cholangiocarcinoma 6 years prior was diagnosed with postoperative cholangitis due to intrahepatic lithiasis. Removal of the intrahepatic stones was attempted using balloon-assisted endoscopy; however, the endoscope could not reach the anastomotic site. The patient underwent duodenojejunostomy and subsequent endoscopic management. The patient was discharged without complications. Two weeks after the operation, the patient underwent endoscopic retrograde cholangiography through the duodenojejunostomy and the intrahepatic lithiasis was removed.

**Conclusions:**

Duodenojejunostomy allows easy endoscopic access to a BEA. Duodenojejunostomy and subsequent endoscopic management may be an alternative treatment option in patients with BEA strictures that are inaccessible via balloon-assisted endoscopy.

## Background

Hepatobiliary and pancreatic surgery for benign or malignant diseases often requires biliary enteric anastomosis (BEA). One of the long-term complications after BEA is stricture, which has an incidence of 2.6–12% [1–8]. BEA stricture often causes recurrent cholangitis and lithiasis formation. It can significantly affect the quality of life and may even lead to life-threatening complications [9]. Treatment options for postoperative BEA strictures or hepatolithiasis include surgical revision, endoscopic treatment, and percutaneous transhepatic management [10]. Endoscopic management of BEA strictures is a minimally invasive procedure. However, reaching the BEA for subsequent endoscopic treatment is technically demanding despite balloon-assisted endoscopy because of the surgically altered anatomy [11]. If the biliary anatomy is inaccessible for endoscopic retrograde cholangiography (ERC), treatment should be performed.

This report describes duodenojejunostomy and subsequent endoscopic management as an alternative surgical technique for strictures of the BEA.

## Case presentation

### Case 1

**Fig. 1:**
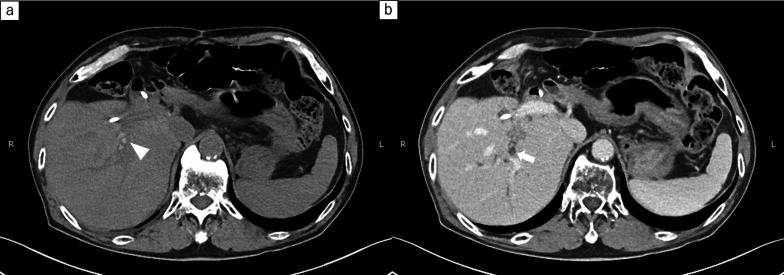
Computed tomography (CT) images of Case 1.** a** Plain CT,** b** Enhanced CT revealed intrahepatic lithiasis (arrowhead) and intrahepatic biliary dilation (arrow)

An 84-year-old man presented with fever and jaundice. Computed tomography (CT) revealed intrahepatic lithiasis (Fig. 1), leading to the diagnosis of postoperative cholangitis due to intrahepatic lithiasis. He underwent left hepatic trisectionectomy for hilar cholangiocarcinoma 6 years prior and suffered from recurrent postoperative cholangitis. In his first session occurring 2 years before admission, his cholangitis due to anastomotic stricture was treated with percutaneous transhepatic biliary drainage in the first session. After percutaneous transhepatic balloon dilation, a biliary stent was endoscopically inserted, and the percutaneous transhepatic biliary tube was removed. He also underwent surgery for small bowel obstruction 1 year prior to admission. First, a stent exchange was attempted. However, balloon-assisted endoscopy could not reach the anastomotic site, and the stent insertion failed. A multiple biliary approach was considered necessary and reduced the quality of life because of the insertion of the percutaneous drainage tube.

**Fig. 2:**
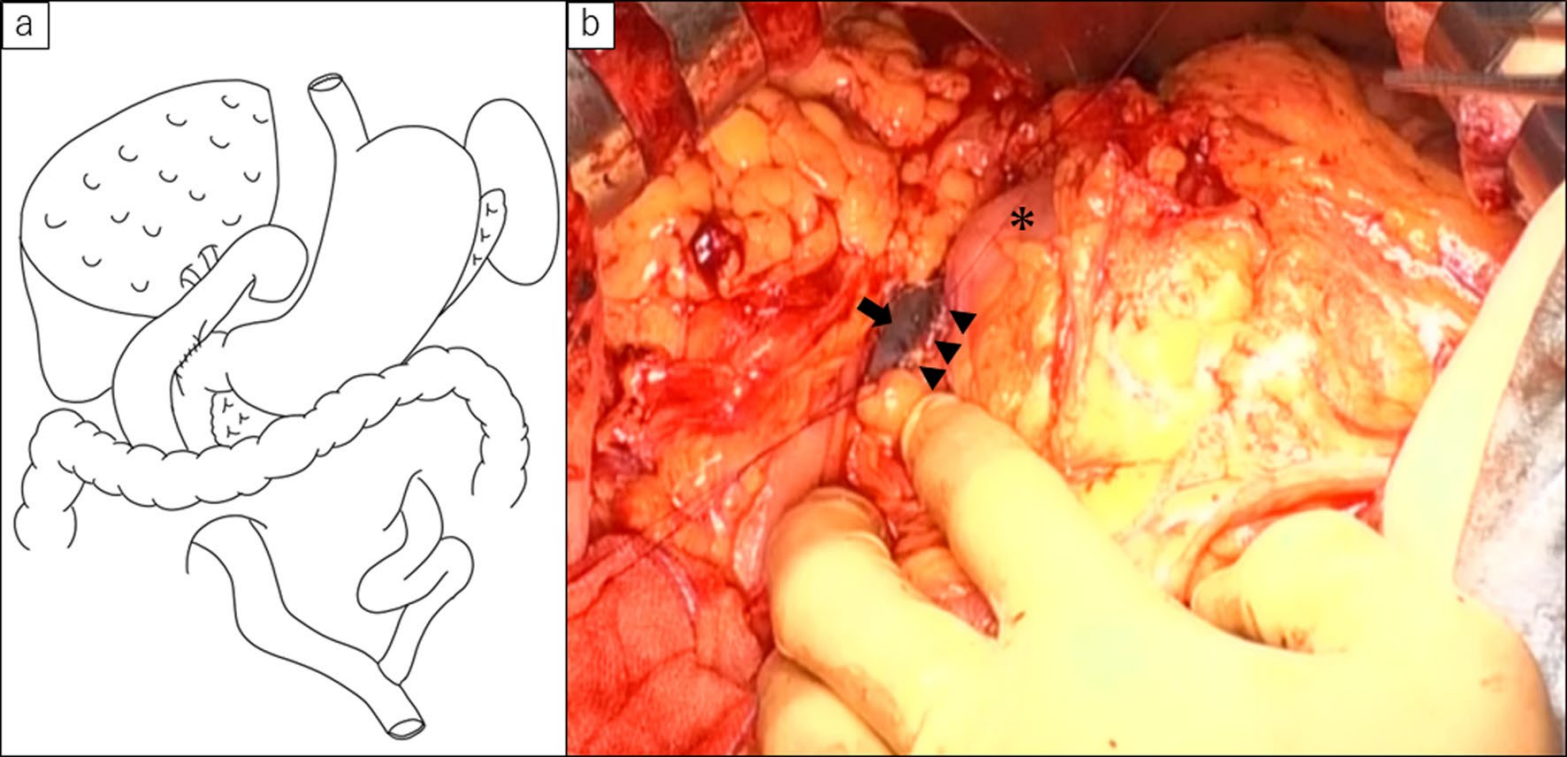
**a** Schematic diagram showing the reconstruction method for Case 1. The patient underwent left hepatic trisectionectomy with Roux-en Y reconstruction. Duodenojejunostomy was added for endoscopic management. **b** Intraoperative photo of Case 1. Arrowhead showing anastomotic site of duodenojejunostomy. The opposite side of hepaticojejunostomy was marked with ink when balloon assisted endoscopy reached the anastomotic site (arrow). Asterisk; antrum of the stomach

**Fig. 3:**
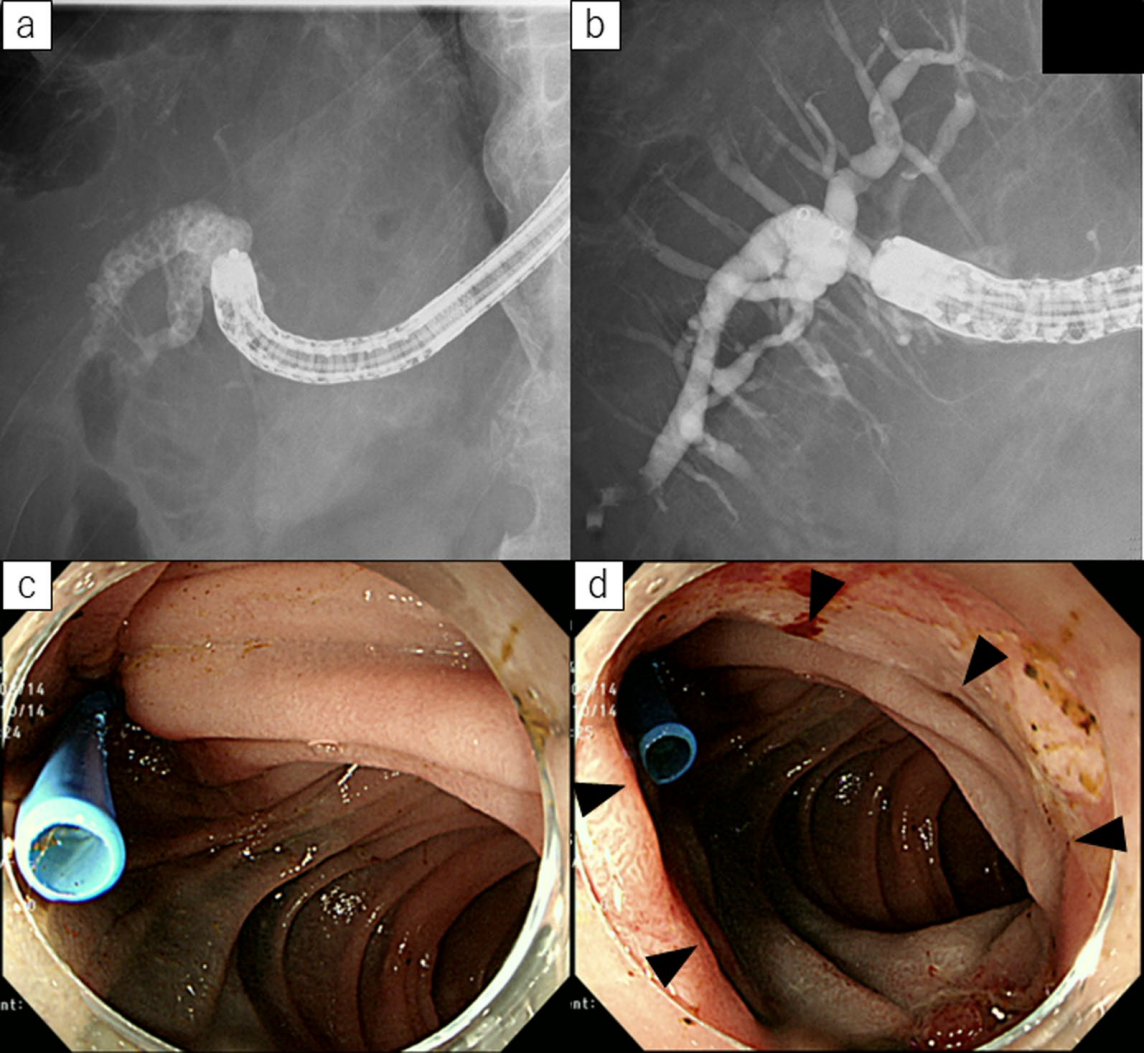
Postoperative endoscopic management for Case 1. **a** Endoscopic retrograde cholangiography (ERC) revealed multiple intrahepatic lithiasis in posterior hepatic duct. **b** After endoscopic lithotomy, intrahepatic lithiasis were completely removed. **c**, **d** Conventional endoscopy could reach biliary enteric anastomotic site through duodenojejunostomy (arrowhead)

After meticulous discussion, the care team decided to perform a duodenojejunostomy to create a biliary access route. Abdominal exploration revealed severe adhesions in the upper abdominal cavity. Adhesiolysis of the right upper abdominal cavity was performed carefully. After the jejunal limb and duodenal bulb were identified, duodenojejunostomy was performed using a side-to-side continuous layer-to-layer suture in 4 cm diameter (Fig. 2). Operation time was 2 h 22 min, and blood loss volume was 80 mL. On postoperative days 8 and 15, he underwent endoscopy to exchange the biliary stent and remove intrahepatic stones. The patient was discharged without any serious complications on postoperative day 18. However, 2 months after the operation, he developed cholangitis and was readmitted to our hospital. During hospitalization, endoscopic management through duodenojejunostomy was performed, and intrahepatic stones were completely removed (Fig. 3).

### Case 2

**Fig. 4:**
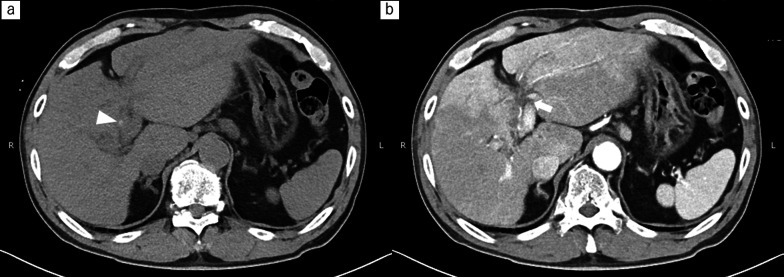
Computed tomography (CT) images of Case 2.** a** CT revealed intrahepatic lithiasis (arrowhead), and intrahepatic biliary dilation (arrow).** b** Early enhancement around the dilated hepatic duct suggested acute cholangitis.

**Fig. 5:**
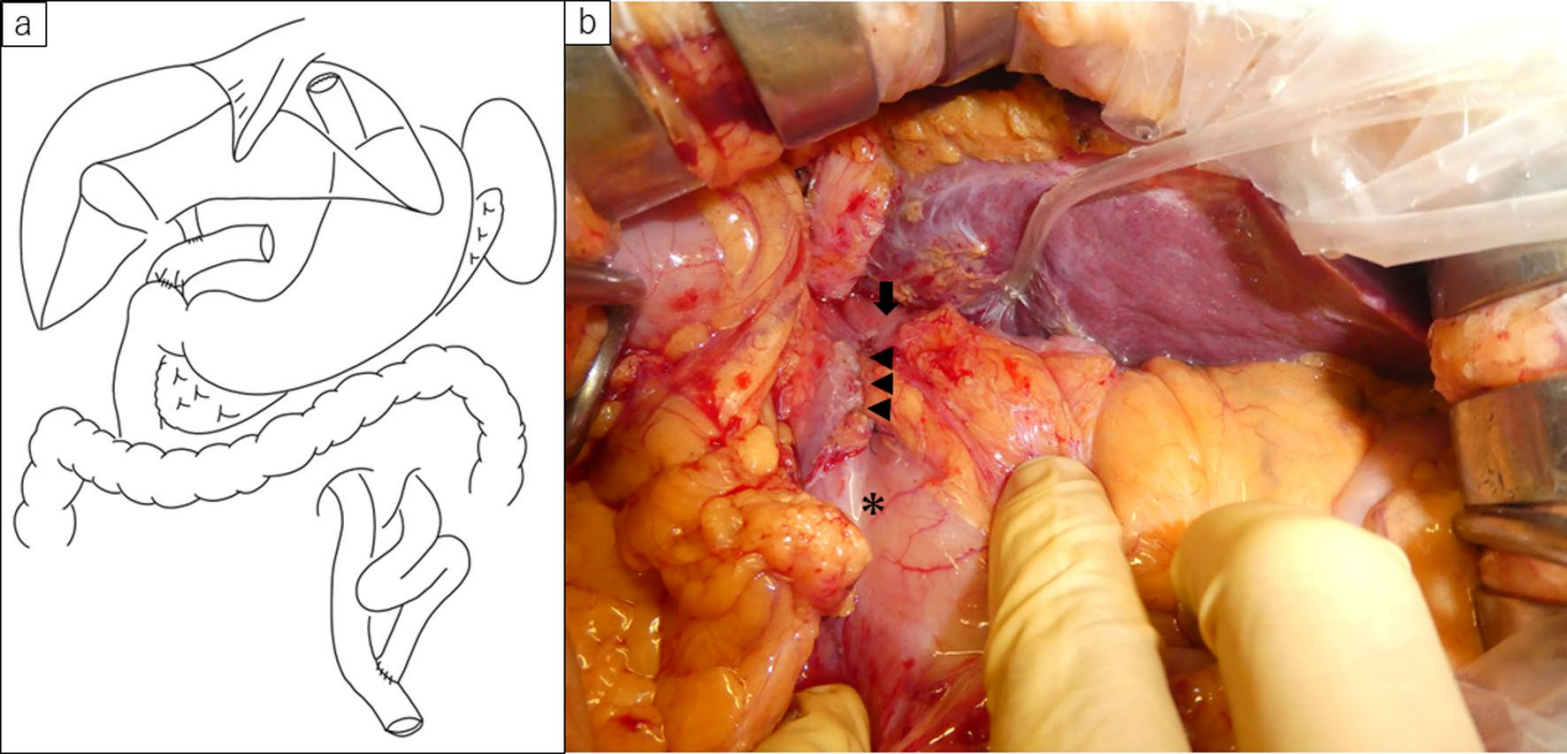
**a** Schematic diagram showing the reconstruction method for Case 1. The patient underwent bile duct resection with retro gastric route Roux-en Y reconstruction. Duodenojejunostomy was added for endoscopic management. **b** Intraoperative photo of Case 2. Arrowhead showing anastomotic site of duodenojejunostomy. Arrow; jejunal limb. Asterisk; antrum of the stomach

**Fig. 6:**
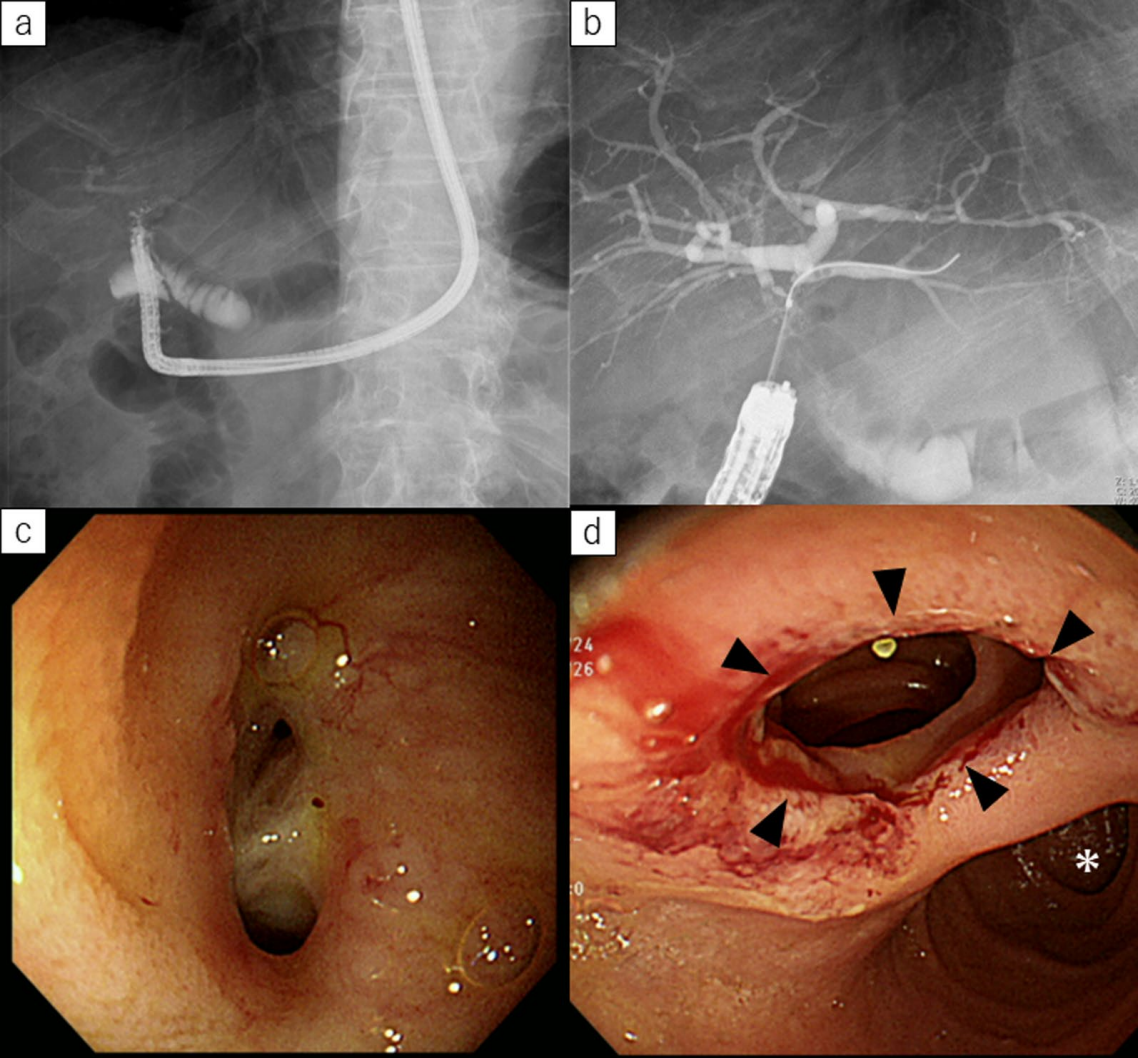
Postoperative endoscopic management of Case 2. **a** Conventional endoscopy could reach biliary enteric anastomotic site. **b** After endoscopic lithotomy, intrahepatic lithiasis were completely removed. **c** Conventional endoscopy could observe biliary enteric anastomotic site. **d** Endoscopy showing duodenojejunostomy site (arrowhead) and descending portion of duodenum (asterisk)

A 75-year-old man presented to our hospital with epigastric pain. Laboratory examination revealed elevated C-reactive protein (12.75 mg/dL), serum bilirubin (2.4 mg/dL), alkaline phosphatase (233 U/L), and gamma-glutamyl transpeptidase (440 U/L) levels. He had undergone bile duct resection for hilar cholangiocarcinoma 6 years prior. He also suffered from cholangitis and intrahepatic lithiasis, which were treated with percutaneous transhepatic balloon dilation for biliary stricture. The percutaneous transhepatic biliary tube was removed after the biliary stricture treatment. CT scan revealed a high-density area in the left hepatic duct and a slightly dilated intrahepatic bile duct (Fig. 4). The patient was diagnosed with postoperative cholangitis due to intrahepatic lithiasis. Removal of the intrahepatic stone was attempted using balloon-assisted endoscopy; however, the endoscope could not reach the anastomotic site. The patient underwent duodenojejunostomy. After adhesiolysis around the jejunal limb, duodenojejunostomy was done using a side-to-side continuous layer-to-layer suture in 4 cm diameter (Fig. 5). to aid subsequent endoscopic management. Operation time was 1 h 3 min, and blood loss volume was 10 mL. The cholangitis improved with antibiotic treatment alone. The patient was discharged on postoperative day 7. Two weeks after the operation, he underwent ERC through duodenojejunostomy to address the intrahepatic lithiasis (Fig. 6).

## Discussion

BEA is a standard operative procedure for patients with various diseases of the hepatobiliary and pancreatic regions. Despite advances in surgical techniques, postoperative morbidity following hepatobiliary and pancreatic surgeries remains high. Postoperative cholangitis is one of the major complications after BEA, with an incidence of 7.7–21.1% [4, 6, 12, 13]. Postoperative cholangitis is often associated with BEA stricture [14].

Various interventions including balloon dilation, stent placement, and reoperation are indicated for biliary strictures [10]. ERC using balloon-assisted endoscopy in patients with Roux-en-Y reconstruction remains challenging because of the long tortuous afferent limb and adhesions [15]. Furthermore, balloon-assisted endoscopy has device limitations, owing to the narrow diameter and length of the working channel. When biliary anatomy is inaccessible for ERC, a percutaneous transhepatic biliary drainage (PTBD) is an alternative option that has been commonly used prior to balloon-assisted endoscopy [16]. However, this approach is often associated with catheter-related complications and a decreased quality of life. Moreover, it cannot be performed in patients without obvious dilatation of the intrahepatic bile duct because of difficulties associated with biliary catheterization [16, 17]. Conventional endoscopy can reach the BEA site in patients who have undergone duodenojejunostomy. As there were no device limitations, stent insertion and stone removal were relatively easy. In the present report, both patients were successfully managed using conventional endoscopy.

Even if the stricture is dilated, anastomotic stenosis may recur [18, 19]. Thus, patients who develop anastomotic strictures sometimes require repeated interventions. As this report demonstrates, creation of a duodenojejunostomy allowed for easier endoscopic access to the BEA and repeat therapeutic ERC. In the present report, although both patients required several sessions of therapeutic ERC, intrahepatic lithiasis was successfully removed.

Recently, endoscopic ultrasonography-guided biliary drainage (EUS-BD) was reported an alternative way for treatment of biliary obstruction when the papilla is not endoscopically accessible [20]. This EUS-BD technique is also indicated for benign biliary stricture in patients with surgical altered anatomy as an EUS–hepaticogastrostomy (HGS) [21]. EUS–HGS were described as a safe and valuable way for biliary drainage with a high technical and clinical successful rate compared with PTBD [22]. However, the biggest limitation of EUS–HGS is the technical difficulty. Because serious adverse event including bile leakage, perforation and migration of the stent may happen, EUS–HGS is available only in a small number of hospitals [23]. While duodenojejunostomy can be performed in many hospitals which have experienced surgeons.

Surgical revision is generally indicated when non-operative management fails. However, the operative revision of biliary strictures is challenging because of dense adhesions and persistent chronic inflammation [24]. Re-anastomosis also carries the risk of developing recurrent strictures [25]. Duodenojejunostomy, which requires only adhesiolysis around the jejunal limb and duodenum, is technically simpler than surgical revision. Some previous reports described an internal anastomosis between the Roux-en-Y limb and the stomach or duodenum, allowing endoscopic access to the BEA [26–28]. Duodenojejunostomy is an acceptable reconstruction technique.

Endoscopic biliary drainage subsequent to duodenojejunostomy is considerable treatment option for stricture of BAE when balloon-assisted endoscopy cannot reach BAE. Duodenojejunostomy can allow easy access to BAE and re-intervention for repeated stricture. This surgical technique is simpler than surgical revision and can be performed many hospitals. Because catheter-related complications do not happen, quality of life may be better than PTCD or EUS–HGS. However, duodenojejunostomy require general anesthesia and may be more invasive compared with PTCD or EUS–HGS. In addition, this reconstruction methods may cause ascending cholangitis due to the presence of food residue and gastric juice directly in the biliary tree. In the present report, no symptoms were observed in either patient within a few months following duodenojejunostomy. Postoperative ascending cholangitis should be carefully considered during follow-up.

## Conclusion

Duodenojejunostomy allows easy endoscopic access of the BEA. Duodenojejunostomy and subsequent endoscopic management may be an alternative treatment option in patients with BEA that is inaccessible using balloon-assisted endoscopy.

## Data Availability

All data generated or analyzed during this study are included in the published article.

## Abbreviations

BEA: Biliary enteric anastomosis
ERC: Endoscopic retrograde cholangiography
CT: Computed tomography
PTBD: Percutaneous transhepatic biliary drainage
EUS-BD: Endoscopic ultrasonography-guided biliary drainage
EUS–HGS: Endoscopic ultrasonography–hepaticogastrostomy
